# The present and future of cardiological telemonitoring in Europe: a statement from seven European countries

**DOI:** 10.1007/s00399-025-01076-8

**Published:** 2025-04-08

**Authors:** Thomas M. Helms, Giuseppe Boriani, Hans-Peter Brunner-La Rocca, Cedric Klein, Friedrich Koehler, Paweł Krzesiński, Yannick Maaser, Anne Neumann, Jose L. Merino, Carsten Schultz, David Jay Wright, Bettina Zippel-Schultz, Gerhard Hindricks

**Affiliations:** 1 German Foundation for the Chronically Ill, Berlin, Germany; 2 Peri Cor Cardiology Working Group/Ass. UCSF, Hamburg, Germany; 3 Cardiology Division, Department of Biomedical, Metabolic and Neural Sciences, University of Modena and Reggio Emilia, Polyclinic of Modena, Modena, Italy; 4 Department of Cardiology, Maastricht University Medical Centre, School for Cardiovascular Diseases (CARIM), Maastricht, The Netherlands; 5 Department of Cardiovascular Medicine, Lille Hospital University, Lille, France; 6 Centre for Cardiovascular Telemedicine, Department of Cardiology, Angiology and Intensive Care Medicine, German Heart Center of the Charité (DHZC), Berlin, Germany; 7 Department of Cardiology and Internal Diseases Military Institute of Medicine-National Research Institute, Szaserow Street 128, 04-141 Warsaw, Poland; 8 La Paz University Hospital-IdiPaz, Autonoma University, Madrid, Spain; 9 Kiel Institute for Responsible Innovation, Kiel University (CAU), Kiel, Germany; 10 Department of Cardiology, Liverpool Heart and Chest Hospital, Liverpool, UK; 11 Department of Cardiology, Angiology and Intensive Care Medicine, German Heart Center of the Charité (DHZC), Charitéplatz 1, 10117 Berlin, Germany

**Keywords:** Cardiac telemedicine, Remote patient monitoring, Digital health technology, Heart disease management, Healthcare innovation, Kardiologische Telemedizin, Telemedizinische Patientenüberwachung, Digitale Gesundheitstechnologie, Versorgung bei Herzerkrankungen, Innovationen im Gesundheitswesen

## Abstract

Cardiovascular diseases remain one of the leading causes of death worldwide, placing a significant burden on individuals, families and healthcare systems. Telemedicine, in particular remote monitoring of patients with cardiovascular diseases, reduces this burden as it links the continuous monitoring of the health status with individual education and adaptation of the therapy to the needs of the patients. This improves patient outcomes and facilitates access to specialised healthcare services, independent of time and distance. Furthermore, telemedicine enables improvements in efficiency and promotes patients’ self-care. However, the widespread adoption of remote patient monitoring faces several hurdles. A round table of experts from seven European countries (France, Germany, Italy, Poland, Spain, the Netherlands and the United Kingdom) reviewed the current state of telemedicine within the participating countries in order to learn from each other with an impetus for European co-operation. The creation of reliable regulations, overcoming regional differences, the redefinition of roles and processes, the personalisation of healthcare services, the promotion of innovation and research, the use of artificial intelligence and, finally, the efficient management and safeguarding of healthcare data were identified as key levers for further development of telemedicine. This discussion paper emphasises the need for cross-national research activities, involving all stakeholders, such as researchers, industry and patients, to foster the integration of telemedicine in clinical pathways.

## Abstract (de)

Herz-Kreislauf-Erkrankungen zählen weltweit unverändert zu den Haupttodesursachen und stellen für die einzelnen Betroffenen, ihre Familien und die Gesundheitssysteme eine erhebliche Belastung dar. Telemedizin, insbesondere die telemedizinische Überwachung von Patienten mit Herz-Kreislauf-Erkrankungen, verringert diese Last, da sie das kontinuierliche Monitoring des Gesundheitszustands mit individueller Edukation und Anpassung der Therapie an die Bedürfnisse der Patienten verbindet. Dies verbessert die Behandlungsergebnisse der Patienten und erleichtert den Zugang zu spezialisierten Gesundheitsleistungen, unabhängig von Zeit und Entfernung. Darüber hinaus ermöglicht Telemedizin Effizienzsteigerungen und fördert die Selbstfürsorge der Patienten. Die breite Umsetzung der telemedizinischen Patientenüberwachung sieht sich allerdings mit einigen Hindernissen konfrontiert. Eine Expertenrunde mit Vertretern aus sieben europäischen Ländern (Frankreich, Deutschland, Italien, Polen, Spanien, Niederlande, Großbritannien) hat die aktuelle Situation der Telemedizin in den teilnehmenden Ländern untersucht, um mit einem Impuls für europäische Zusammenarbeit voneinander lernen zu können. Die Schaffung verlässlicher Regelungen, das Überwinden regionaler Unterschiede, die Neudefinition von Rollen und Abläufen, die Personalisierung von Gesundheitsleistungen, die Förderung von Innovation und Forschung, die Anwendung künstlicher Intelligenz und schließlich die effiziente Gesundheitsdatenverwaltung und der Datenschutz wurden als entscheidende Ansatzpunkte für die Weiterentwicklung der Telemedizin identifiziert. Das vorliegende Diskussionspapier betont die Notwendigkeit länderübergreifender Forschungsaktivitäten unter Einbeziehung aller Interessenvertreter, beispielsweise von Forschern, Industrie und Patienten, um die Integration der Telemedizin in klinische Behandlungspfade zu fördern.

## Introduction

Cardiovascular disease (CVD) is one of the leading causes of death in Europe and worldwide, placing a significant burden on society and healthcare systems [1]. The prevalence of CVD has risen sharply due to an ageing population, unhealthy lifestyle and an increasing number of risk factors. Currently, more than 85 million people in Europe are affected, and this number continues to grow [2]. Access to specialist care is often limited, with long waiting times and considerable differences in care provision between countries as well as between urban and rural regions [3, 4].

Telemedicine, especially remote patient monitoring (RPM), is becoming a key element in cardiological care, changing patient care and healthcare systems across Europe. Current telemedicine applications include: (a) structured telephone support, e.g. calls from heart failure (HF) nurses [5]; (b) non-invasive telemonitoring of pre-specified parameters, e.g. weight, blood pressure, ECG, quality of life or other patient-reported outcome measures [6, 7]; (c) invasive telemonitoring via cardiac implantable electronic devices (CIEDs), e.g. implantable cardioverter defibrillator (ICDs) or cardiac resynchronisation therapy (CRT) [8, 9]; and (d) invasive monitoring through implanted sensors, e.g. measuring pulmonary artery pressure [10]. Future telemedicine solutions will further improve and simplify care, offering benefits such as continuous monitoring, personalised treatment, better patient engagement, improved access to specialised care as well as reduced hospitalisations and mortality [11]. However, widespread adoption still faces significant challenges [12].

A roundtable of 15 experts in cardiology, technology and innovation management and health sciences from seven European countries (France, Germany, Italy, Poland, Spain, the Netherlands and the United Kingdom [UK]) as well as industry representatives was convened to evaluate and compare the status quo of telemedicine, especially RPM, in different European countries and share visions about solutions that go beyond the status quo. The goal was to identify best practices to foster synergies in Europe and learn from each other’s experiences. Key factors influencing the future development of telemedicine were identified and possible solutions discussed.

This article summarises in an exemplary manner the status quo of cardiological telemedicine, especially RPM, in the seven European countries including regulatory, administrative, technological and societal challenges. With this article, the authors want to point out necessary steps that will have a significant influence on future developments and thus on the long-term introduction and sustainable implementation of telemedicine.

## Differences in healthcare systems across Europe

The availability of medical services depends heavily on the regulatory and financial framework of each healthcare system. Two main types can be distinguished: (1) a national health service, funded through tax revenue (Italy, Spain and the UK), and (2) a social insurance system, primarily financed through statutory health insurance (France, Germany, the Netherlands and Poland). Some countries, e.g. the Netherlands, combine a social insurance system with private insurances. In the Netherlands, social insurance covers basic health services. Additionally, individuals can choose supplementary insurance by private insurance companies. Healthcare system structures also vary considerably at national and regional levels:

- Centralised systems such as those in France and Poland have strong government oversight. In Poland, for example, the Ministry of Health plays a leading and proactive role.
- The National Health Service (NHS) in the UK operates as four largely independent organisations (England, Wales, Scotland and Northern Ireland), each reporting directly to the Department of Health.
- Decentralised systems, such as Italy and Spain, delegate healthcare management to regional authorities. The regions distribute funding to healthcare facilities and coordinate healthcare organisation and provision. The national government only defines a catalogue of health services and coordinates between the individual regions. This fragmentation results in inconsistent requirements, processes and standards.
- Germany has a complex healthcare system involving multiple stakeholders responsible for organisation, administration and financing. While the Ministry of Health sets the regulatory framework, the Federal Joint Committee (G-BA) is responsible for organisation and reimbursement. The biggest hurdle of the German healthcare system is a strict separation of outpatient and inpatient care, which is unique in an international comparison. This creates barriers, especially for services that span different sectors, such as telemedicine.

Table 1 provides a summary and an excerpt from the characteristics and challenges of the healthcare systems under consideration.

**Table 1:** Excerpt from the characteristics and challenges of the healthcare systems under consideration

Country	Healthcare model	Funding source	System structure	Key challenges
France	Social insurance system	Statutory health insurance	Centralised, Ministry of Health plays major role	Strong centralisation, administrative challenges
Germany	Social insurance system	Statutory health insurance	Fragmented structure with multiple stakeholders	Coordination of stakeholders, separation of outpatient and inpatient care limits integration
Italy, Spain	National health system	Tax-funded	Decentralised; regional authorities manage funds and services	Regional disparities, inconsistent standards across the regions; unequal distribution of resources
Poland	Social insurance system	Statutory health insurance	Centralised, very active Ministry of Health	Strong centralisation, bureaucratic and administrative challenges
The Netherlands	Mixed system (social and private insurance)	Statutory health insurance + private insurance	Social insurance covers basic care; optional private insurance	Balancing public and private healthcare roles
United Kingdom	National Health System (NHS)	Tax-funded	Four largely independent organisations (England, Wales, Scotland and Northern Ireland)	Regional disparities, NHS budget constraints

## Status quo of telemedicine in selected European countries

### France

In 2014, the French Social Security Financing Act No. 2013-1203 was introduced to promote the telemedicine and improve healthcare. In the ÉTAPES programme for the promotion, development, evaluation and reimbursement of telemedicine, solutions for cardiac, renal and respiratory failure were tested and extended to all regions by Law No. 2016-1827, and to CIEDs and diabetes mellitus in 2017. In 2023, the reimbursement of teleconsultations and tele-expertise by the statutory health insurance scheme was transferred into common law. Tariffs for remote monitoring were included into the compulsory health insurance. It contains teleconsultation (patient to HCP), tele-expertise (exchange of expert opinions), remote medical monitoring and interpretation of patient data, remote assistance between healthcare providers (HCPs) and medical advice by telephone. For remote medical monitoring, reimbursement requires an operator, a therapeutic training programme and a technical solution declared as medical device^1^. CIED monitoring is reimbursed, including a fixed rate per HCP. Future reimbursement is expected for next generation implanted loop recorders (ILRs—currently not reimbursed). Remuneration for RPM is provided in two packages: one for the operator and one for the manufacturer (Article R. 162-95 II and III of the Social Security Code). It can be considered standard care for CIED patients. For HF patients, inclusion criteria (hospitalisation for acute HF in previous 12 months, NYHA class ≥ II and BNP (brain natriuretic peptide) >100 pg/ml or NT-pro BNP >1000 pg/ml) and level of reimbursement appear to be in favour of the future development of RPM [13]. The current “List of reimbursable products and services” is set to be continued for RPM^2^. Synergies and economies of scale are expected, so that the tariffs for medical device manufacturers will be degressive with the number of patients treated.

### Germany

Randomised controlled trials (RCTs) in Germany have shown the clinical and economic benefits of RPM complementing guideline-based therapy, e.g. for HF patients [6, 8, 14]. Nonetheless, implementation has proven challenging. As RPM is a complex holistic care intervention incorporating hospitals and various HCPs, it must be carefully adapted to the characteristics of the German healthcare system with its strict separation of the outpatient and the inpatient sector, including different reimbursement rules. In 2019, the German Cardiac Society defined basic quality requirements for telemedical centres to drive implementation and quality standards [15]. In 2020, the G‑BA approved the reimbursement of a data-supported, timely management for HF patients by statutory health insurance funds^3^. The covered telemedical outpatient services are available for the following patients: NYHA class II or III, left ventricular ejection fraction (LVEF) < 40%, CIED (ICD; CRT‑D or CRT pacemaker [CRT- P]), or a 1-year history of HF hospitalisation, and guideline-based treatment. By 2022, approximately 200,000 HF patients met the inclusion criteria and were eligible for RPM [16]. Reimbursement is now organised via the statutory outpatient remuneration system. It includes diagnosis, instructions and information of the patient, telemonitoring by means of implanted or external measuring devices, a surcharge for intensified monitoring and a flat-rate fee for the external measuring devices. Notable future telemedical solutions are, e.g. developed by the project Telemed5000, which aims to develop an RPM system that is supported by artificial intelligence (AI) and enables telemedical co-care for thousands of high-risk cardiological patients. The project builds on the 5‑year Fontane study that showed positive effects on the lives of HF patients and a reduction in the number of days spent in hospital [6].

### Italy

Italy’s regionalised healthcare system makes the implementation of telemedical services challenging. Still, RPM can be considered close to a standard of care for device checks of CIEDs. It is currently regulated and reimbursed in 11 out of 22 regions and the tariff corresponds to an in-office check, with maximum four requests for the pre-defined reimbursement per patient and year [17]. Remuneration is generally low and covers only device checks, not the disease management itself [18]. There remain problems related to personnel resources and costs for technical transmitter devices. Between 2012 and 2017, the number of patients treated per centre has increased. This trend was reinforced by the COVID-19 pandemic [19]. Nevertheless, implementation of RPM in practice is largely dependent on initiative and willingness of local cardiologists and teams of nurses and technicians. The planned centralisation of RPM in Italian centres has not been followed through and there is a lack of official plans for large-scale implementation. The use of RPM for disease management in HF, which requires commitment, dedicated staff and appropriate organisation, is variably adopted due to logistical challenges and the lack of appropriate reimbursement. Current research in Italy includes the integration of smartphone applications (apps) into the RPM of HF patients with CIEDs. The results indicate high access and acceptance, especially in young and highly educated patients. Older patients, in turn, show higher long-term adherence [20].

### Poland

In 2022, the Polish government set up a roundtable for telemedicine with around 100 experts, and included telemonitoring in the National Cardiovascular Disease Programme for CVD. Polands e‑health landscape includes medical records, e‑patient accounts, e‑referrals and e‑prescriptions as well as various telemedicine services. Currently, hybrid cardiac telerehabilitation (HCTR), RPM for patients with CIEDs and ILRs are being offered and reimbursed. Non-invasive ambulatory long-term ECG monitoring is reimbursed. However, services with more than 7 days of monitoring are still underfinanced. HCTR has been available since 2018 for patients after acute coronary syndrome and valve surgery. It is limited to 90 days, consisting of the initial stage in an outpatient clinic or hospital ward and a second stage of telemonitored training sessions at home. Each training session is reimbursed individually with a capped flat rate for up to 24 sessions. HCTR shows high adherence and is associated with improved prognosis for mortality and hospitalisations [21, 22]. RPM of CIEDs is performed via outpatient cardiology clinics with experience of a minimum of 100 implanted ICD/CRT‑D. The service is available to HF patients with reduced LVEF, having an ICD or CRT‑D and to patients <18 years with an implanted pacemaker/CRT. It includes the device function, relevant cardiovascular events, ICD shocks and, optionally, HF status. Data analysis is performed during working hours, leading to teleconsultation within 24/72 h in the case of a clinically justified event. Remote device checks are performed at least three times a year and a personal consultation at least once a year. Reimbursement for RPM of CIEDs consists of a fixed fee for the transmitter, consultation with instruction and education of patients as well as a yearly flat fee for the telemonitoring service. RPM for ILR is set to be reimbursed for patients after cryptogenic stroke or with recurrent unexplained loss of consciousness. Non-invasive ambulatory long term ECG monitoring may be performed using multiple different Holter ECG systems. Other ECG modalities, such as event ECG recorders, patches, and belts [23], are offered only by some private medical service providers. Non-invasive RPM is promoted by the Ministry of Health by programme “Telemonitoring of persons with heart failure”, remote platform of home care “DOM”, e‑consultation platform programme and public funding of trials, i.e. TELEREH-HF [22], NOMED-AF [24] and AMULET [25], combining nurse-led ambulatory care points with remote cardiologist support, employing home telemonitoring and individualised hemodynamic assessments for HF patients [26]. AMULET demonstrated promising results in the reduction of cardiovascular mortality and hospitalisations [27].

### Spain

Spain’s regionalised healthcare system and lack of a unified institutional and regulatory frameworks hinder RPM implementation [28]. In 2003, the Spanish government defined the “National Interoperability Framework”, a catalogue of criteria for security, standardisation, storage and formats of healthcare data. It is based on an agreement between the 17 autonomous regions and should be updated regularly. In 2010, Royal Decree 4/2010 established criteria and specific principles that define interoperability in the public sector and therefore also in the healthcare sector. Already in 2011, around two thirds of Spanish hospitals had an EPR. However, implementation within individual regions differs. Currently, there is no remuneration for RPM in Spain. Implementation of RPM suffers from substantial regional differences. As described in Italy, the number of RPM activations during COVID-19 showed a significant increase, totalling in a major increase in CIED remote monitoring and in RPM activations [29]. The general trend towards digital services is also seen in Spain, with service providers for teleconsultation in remote outpatient hospitals growing, a cooperation between the telecommunications company Telefónica with an US telemedicine provider and a 24/7 availability of Movistar Salud providing advice to patients with the aim of reducing hospital visits^4^. However, these generic counselling services cannot meet the HF-specific needs of an RPM, like HF follow-up and ECG telemonitoring, and therefore require further expansion.

### The Netherlands

The Netherlands has a well-developed e‑health landscape including e‑prescriptions, a patient portal, remote consultations, telemonitoring and EPRs in primary, secondary and tertiary care. In addition, teleconsultation has been established in outpatient care for more than two decades. The Dutch government promotes telemedicine and e‑health solutions with a large amount of funding. However, data exchange between HCPs is hampered by the variety of more than 3000 regional EPRs that are connected to the national digital infrastructure AORTA [4]. The need for standardisation has been identified but progression is slow and implementation currently still lacking. Telemonitoring in cardiology was already introduced in the 2000s but gained a significant increase in interest only during COVID-19. The Council for Public Health and Society introduced a classification of digital care including, e.g. e‑diagnosis, e‑consultations, e‑care such as monitoring, e‑prevention and intervention in high-risk individuals. Digital healthcare services that have proven safety and effectiveness and meet the ‘package criteria’ can be declared for coverage under the Health Insurance Act and Long-term Care Act. The Health Insurance Act outlines which medical devices are covered by the basic health insurance package. Some services fall under specialised care and are hospital-billed, while others are reimbursed by insurers as medical device care. Reimbursement has been structurally available for telemedicine for specialised care since 2023^5^, which also includes remote interrogation of CIEDs. Telerehabilitation is partly reimbursed. Still, implementation is rather scarce in clinical practice, characterised by regional differences. The most popular type of RPM is the telemonitoring of HF or AF patients, including the control of symptoms, blood pressure, lifestyle, risk behaviour and to a limited extent links with other chronic diseases. Invasive pulmonary artery pressure monitoring in HF (CardioMEMS) has been reimbursed conditionally to participation in a randomised controlled trial [30] as a novel model to test the impact on national implementation of new expensive care pathways. Structural reimbursement is pending. Furthermore, there are various local telemedicine initiatives, like “The Box” providing patients with telemedical tools and apps. It uses telemedicine in the outpatient follow-up of patients, e.g. with acute myocardial infarction [31].

### United Kingdom

Between 2001 and 2019, UK predominantly targeted long-term conditions such as COPD, HF and diabetes mellitus for RPM [32]. In care homes, early intervention strategies were implemented to alleviate the economic burden^6^. The onset of the COVID-19 pandemic necessitated a reprioritisation towards telemedicine, e.g. Covid Oximetry @home programme [33]. In 2021 and 2022, the focus shifted towards elective recovery of the backlog caused by the pandemic, with technology being reconfigured for triaging waiting lists^7^. The introduction of virtual wards for several indications, including HF, enabled step-down care, allowing early hospital discharge and reducing lengths of stay, and step-up care after GP referral^8^. By 2022 and 2023, RPM applications were expanded to include the management of long-term conditions, enabling care at home concepts and other out-of-hospital services [34]. By October 2023, NHS England provided 10,421 virtual beds and over 240,000 patients had been treated in virtual wards^9^.

Many telemonitoring devices and services are available and reimbursed in the UK. Interventions have multifaceted action points, from prevention of admissions, through primary care management and peri-operative management in secondary care, to RPM, e.g. for patients with CIED [35]. However, implementation of teleservices is patchy and requires a service reconfiguration. Especially the management of associated data presents a considerable challenge. There is an anticipation for the integration of RPM with shared EPRs and population health analytics, expanding the scope to encompass wellness, passive monitoring for conditions such as frailty and social care^10^. As a publicly funded healthcare system, the NHS offers different incentives, flexibility and opportunities compared to other systems [4]. Overall, the UK is making good progress in integrating telemedical services into a holistic health management approach, even though the velocity of adoption seems to have slowed down again after the pandemic.

Table 2 summarises the described status of telemonitoring and its reimbursement in the seven countries, the associated barriers and exemplary prospects.

**Table 2:** Status of telemonitoring and its reimbursement in the seven countries, the associated barriers and exemplary prospects

Country	Telemedicine policy	Key reimbursed services	Challenges	Future outlook
France	ÉTAPES program for telemedicine reimbursement; integrated into statutory health insurance since 2023	Teleconsultation, tele-expertise, RPM for HF, diabetes and CIEDs	Inclusion in list of reimbursable products and services	Expected expansion of reimbursement to ILRs, scalable reimbursement models
Germany	RPM reimbursed for HF patients under statutory health insurance	HF RPM covering telemonitoring, device management, patient education	Strict outpatient–inpatient separation complicates implementation	Expansion of AI-supported RPM solutions like Telemed5000
Italy	RPM for CIED checks reimbursed in 11 out of 22 regions	CIED checks (max. 4/year), limited coverage for disease management	Regional variation, lack of disease management reimbursement	Speed up national expansion, current projects focus, e.g. on wearable integration for HF RPM
Poland	Telemedicine included in the National CVD Program	Hybrid cardiac telerehabilitation (HCTR), long-term ECG monitoring, RPM for CIEDs and ILRs	Underfunding for long-term ECG monitoring	Strengthening of nurse-led telemedical care models
Spain	Regional disparities hinder national RPM implementation	Limited reimbursement, significant regional variation	Significant regional disparities in telemedicine access	Gradual growth in teleconsultation services, but HF-specific RPM needs expansion
The Netherlands	Strong e‑health system with telemedicine funding	Telemonitoring for HF and AF patients, CIED interrogation, telerehabilitation is reimbursed partly	Lack of standardized EPRs slows integration	Speed up implementation of new technologies, potential for broader RPM and AI integration
United Kingdom	NHS supports RPM, virtual wards and telehealth	RPM for chronic conditions (COPD, HF, diabetes); virtual wards expanding to long-term care	Patchy implementation, need for better RPM data integration	NHS aiming for increased RPM integration with shared EPRs and population analytics

## Shared challenges and opportunities in the implementation of telemedicine

The rapid expansion of digital devices, smartphones and the internet of things (IoT) offers significant opportunities for the advancement of telemedicine in cardiovascular care [36]. This technological process, combined with improvements in internet networks, has created new possibilities for comprehensive CVD monitoring.

Despite promising results from several meta-analyses showing improvements through telemedicine, its clinical application is still limited. This applies in particular to the AI-supported treatment of patients with cardiovascular diseases. The implementation of telemedicine into daily practice faces several common challenges, including regulatory hurdles, data interoperability and reimbursement structures.

To address these collective challenges, European initiatives have emerged alongside national efforts. One example is the PASSION-HF project (funded by InterregNWE 702), an EU consortium led by the Netherlands, which developed and tested a virtual doctor. This system functions as a therapy companion for patients and as a decision support system for HCPs [37, 38]. Additionally, the iCARE4CVD project aims to optimize cardiovascular care by personalising prevention and treatment through early diagnosis, individual risk stratification and AI-based clinical decision-making (Innovative Health Initiative Joint Undertaking [IHI JU] under grant agreement No. 101112022, www.icare4cvd.eu). These exemplary European projects provide important insights into the potential and limitations of telemedicine, forming a valuable basis for further research and evidence.

Based on the results of the joint discussion on the current situation and future challenges, the following aspects were identified as especially important for the future development of telemedicine.

### Establishing reliable regulations: adapting approval and reimbursement norms to the needs of telemedicine

A clear and transparent legal framework with well-defined evaluation and reimbursement processes is essential for the adoption and dissemination of telemedicine. Systems such as the DiGA procedure^11^ in Germany or the Polish Health Application Portfolio (PAZ) serve as examples of structured regulatory pathways. The Dutch model provides a good example by defining quality standards for telemedical services.

Providers must rely on certification procedures and the prospect of profits to develop and offer innovative products/services. At the same time, administrative hurdles for the approval of new products/services must be minimised to maintain a balance between innovation and organisational risk. Currently, in Germany and France, the pathway for telemedical innovations from prototype to market approval and reimbursement can take up to 10 years—as the example of CardioMems shows [39, 40]. Such delays rendering products obsolete by the time they reach market, leading to economic disadvantages and potentially exposing patients to delayed access to clinically beneficial technologies.

The European Medical Device Regulation 2017/745^12^ provides a harmonised framework for innovation approval. However, its requirement for extensive clinical data significantly increases costs and time. Given the rapid evolution of digital health technologies, it is crucial to continuously evaluate the effectiveness of digital solutions and refine the accuracy, e.g. of prediction models [41]. Thereby, it is important to focus not only on products or devices but also on disease management processes and the entire patient care pathway. Reimbursement strategies must be carefully aligned with the structure of each healthcare system, ensuring that funding mechanisms support the full continuum of care rather than focusing on isolated products or interventions.

### Making telemedicine fair: overcoming regional differences and social milieu-based discrimination

The implementation of telemedicine across European countries remains fragmented and inconsistent. Structural deficits, particularly in digital infrastructure and the scalability of telemedicine services, further limit widespread adoption. Rural areas, which could benefit the most from telemedicine, often remain underserved, unable to fully leverage technological advancements. To bridge this gap, targeted support mechanisms are needed, such as collaborations between rural healthcare providers and clinical centres to facilitate integration.

Beyond infrastructure challenges, clinical research and patients’ access to telemedicine are often subject to selection biases, limiting representativeness and generalizability. Many studies are conducted in socio-economically advantaged settings, resulting in outcomes that may not reflect real-world patient populations. Furthermore, patients from lower socio-economic backgrounds and migrant communities are frequently underrepresented in telemedicine programs.

Additionally, barriers include limited access to digital devices, a lack of digital literacy and insufficient technical support. To ensure equitable access, it is essential to integrate diverse patient populations into clinical validation studies and in the implementation of telemedicine programmes. Telemedicine solutions must be accessible and user-friendly for all patients, ensuring that technological advancements translate into tangible improvements in healthcare equity.

### Increasing user orientation: designing the change of roles, responsibilities and processes

The development of digital health solutions often fails to adequately consider the needs, workflows and requirements of potential users, including HCPs, patients and informal caregivers. As a result, acceptance remains low, and many innovations do not reach clinical practice or are introduced after a long delay. To ensure practical applicability, the development of digital health solutions should be driven by the clinical needs, real-world workflows and patient care pathways. By aligning solutions with these fundamental aspects, unrealised potential can be leveraged at the process level [38].

There is an urgent need for telemedicine technologies that are intuitive, adaptable and responsive to individual requirements—including linguistic, cultural or cognitive differences. A co-creation approach, involving device manufacturers, clinicians, patients and researchers from various disciplines, is recommended to enhance acceptance and usability. Such collaborations should be built on trust-based partnerships, ensuring that solutions address real-world challenges rather than technological possibilities alone.

Beyond usability, implementation challenges must be identified and proactively managed. Telemedicine fundamentally reshapes roles within the healthcare system. Patients will become more involved in self-management, while HCPs will be increasingly supported by decision-support systems, enabling them to address a broader range of medical issues, referring only complex cases to specialists. This transformation, however, may pose threats to the professional identity, including concerns over a loss of expertise, changes in reputation and diffusion of responsibilities. These aspects must be acknowledged and actively addressed during the development and integration of digital care models [42].

Finally, the medical technology industry is undergoing a profound shift. With the increase in private health data, manufacturers must expand their responsibilities beyond product sales to include data protection, privacy and ethical considerations. This transforms them from device suppliers into service providers, opening new data-driven business opportunities that can improve patient outcomes but also require new competencies.

### Personalising medicine: increasing the fit of telemedicine with individual patient needs

Patients have individual medical and psychological needs, socio-economic background, cognitive abilities and motivation for self-care. High quality, accessible and efficient telemedicine services need to take these factors into account, as a one-size-fits-all approach is unlikely to be effective. A deeper understanding of patient motivation, support needs and engagement strategies is essential. Telemedicine technology must be meaningful and beneficial to patients—outcomes must be noticeable, and devices must not evoke concerns about privacy. Furthermore, technology should be as unobtrusive and integrated as seamlessly as possible into daily life. Patient education and training are critical to ensure successful adoption and effective use of those technological solutions.

The role of patients in their healthcare journey is evolving, making patients’ self-management skills increasingly important. AI-supported digital solutions that provide personalised, timely and guideline-compliant support will gain importance in future care models. Furthermore, telemedicine in cardiology should focus more on prevention and risk stratification, using predictive analytics to enable early intervention and personalised patient management. These technologies have the potential to drive positive behaviour change, further improving cardiovascular outcomes.

To accelerate the adoption and implementation of clinically validated telemedicine solutions, the identification of patients with the greatest potential benefit should be prioritised. Providing underserved populations with accessible and appropriate technologies is key to achieving meaningful clinical impact. These patient groups can serve as visible examples within their communities, fostering wider acceptance of telemedicine and enabling the collection of high-quality real-world data. Such data is crucial for understanding user behaviour, patient needs, appropriate response mechanisms, pattern recognition and predictive value evaluation—all of which are essential for the further development of AI-driven telemedicine solutions. To facilitate these advancements, comprehensive data collection, structuring and analysis are necessary to ensure continuous refinement and improvement of digital healthcare models.

### Accelerating the diffusion of telemedicine: building evidence through research and forming new alliances for innovation

The participants agreed that despite significant progress in telemedicine for CVD across all countries, current research and innovation efforts must be intensified to establish a robust evidence base for its benefits. Scientific validation remains the key driver of user acceptance—both among HCPs and patients—as well as for the integration and reimbursement of telemedical solutions. Although studies demonstrate promising results, further research is needed to gain additional and broader insights, such as which telemedicine interventions are most effective for a specific patient group and which factors influence patient adherence.

Moreover, the wealth of available medical data remains underutilised. The large amounts of data available, especially from healthcare providers like hospitals or outpatient facilities, and industry hold valuable insights into long term treatment effects, particularly in the context of telemedicine. This data could serve as a foundation for AI-driven prediction models, optimising personal care strategies and helping to identify relevant innovation areas.

To fully leverage these opportunities, public–private partnerships should be expanded. Such collaborations can enhance the knowledge pool, foster innovation and could help to overcome limitations in public funding for research. Establishing cooperation models between medical professionals, industry stakeholders, researchers and funding bodies will be essential to drive the future development and sustainable implementation of telemedicine solutions.

### Making telemedicine more intelligent: use of AI to support healthcare

The importance of AI in healthcare is increasing, offering new possibilities for clinical decision-making, resource optimisation and patient engagement. AI has the potential to support HCPs by tailoring treatment plans to individual patient needs, dynamically adjusting medications and interventions based on real-time responses. This can lead to improved clinical outcomes and more efficient use of resources. For example, AI-driven analysis using Cardio Explorer® can accurately assess the pretest probability of coronary artery disease (CAD), enabling precise risk stratification and ensuring patients are directed to the most appropriate treatment pathway.

The development of advanced algorithms is crucial to make RPM more efficient, cost effective and scalable, as is also being tested in the Telemed5000 project mentioned above [43]. AI models can analyse vast amounts of real-time data while integrating information from various sources, including EPRs, wearable devices and patient-reported outcomes. By synthesising these data sources, AI can provide a comprehensive assessment of a patient’s health status, identifying patterns that indicate early signs of deterioration. This enables predictive alerts and facilitates early intervening before the health condition worsens.

Beyond diagnostics and monitoring, AI-driven solutions can enhance therapy adherence by actively engaging patients in self-management [44]. AI-solutions could autonomously monitor, interpret and even initiate interventions based on continuous patient data input. The above mentioned PASSION-HF project developed and tested a virtual doctor to address these issues. This supports a shift toward decentralised healthcare, where patients can receive high-quality telecare within the comfort of their homes.

However, implementation of AI in healthcare presents challenges and concerns. To ensure safe and effective use, structured education and training are essential, incorporating general and country-specific expert perspectives. National frameworks, such as the Polish White Paper on AI in Clinical Practice [45], provide valuable guidance for addressing these challenges and establishing trustworthy AI applications in medicine.

### Making data for telemedicine safe: interoperability, data management and security

Data is fundamental to diagnosis, treatment and decision-making, particularly in the development and optimisation of AI-driven solutions. However, health-related data is fragmented, usually non-uniform and difficult to access, limiting its potential for improving patient care. Syntactic and semantic interoperability is essential to enable efficient data exchange, seamless cooperation and standardised processing. A key aspect is the harmonisation of data from different vendors—similar to the DICOM standard, which is actively pursued by the European Heart Rhythm Association in cooperation with the World Forum of CIED^13^. Moreover, data federation standards are necessary to facilitate the integration and analysis of data from multiple sources, e.g. as demonstrated in the iCARE4CVD project.

A promising framework for the structured use of data in healthcare is the European Health Data Space (EHDS). EHDS establishes rules, standards, digital infrastructures and governance models, encompassing all stakeholders, such as patients, researchers and clinics, policymakers and industry. It defines guidelines for data provision, accessibility and research applications^14^.

Telemedicine relies on two primary ways of entering data: 1) active input: from patients or HCPs, or 2) passive input: via medical devices that continuously transmit data. A critical challenge is ensuring that transmitted data is accurate, validated and correctly recorded. Only high-quality, reliable data allows meaningful AI-driven processing, precise interpretation and effective integration with other data that may already be available in the EPR. In addition, data security and privacy protection are of highest importance. The transmission and storage of sensitive medical data must adhere to strict security standards to prevent unauthorised access and misuse. Innovative approaches are therefore required to validate the plausibility of collected data and facilitate secure data merging. By addressing these challenges, telemedicine and AI can fully leverage data-driven insights, improving patient outcomes and healthcare efficiency.

## Conclusion

The overview of telemedicine implementation in seven European countries highlights the current state and diverse challenges associated with its implementation into cardiovascular care. Despite significant differences in healthcare systems, regulatory frameworks and adoption rates, the collective experiences underscore that telemedicine has the potential to transform cardiovascular care. The roundtable discussions identified key factors for the advancement of telemedicine, including:

- Regulatory incentives and reimbursement strategies to facilitate broader adoption
- Addressing regional disparities in healthcare delivery and telemedicine infrastructure
- Redefining roles and workflows within healthcare systems to accommodate digital care models
- Personalising healthcare services to meet individual patient needs through a patient-centred approach
- Fostering innovation and research to refine telemedical applications
- Leveraging AI to enhance healthcare delivery and decision support
- Ensuring robust healthcare data management and security to protect patient privacy while enabling seamless interoperability

Furthermore, joint quality standards should be developed to ensure high-quality telemedical care, including standardised provider qualification and service quality benchmarks.

These insights pave the way for a more integrated, efficient and personalised healthcare landscape. They also emphasise the need for collaborative research efforts at both the EU level and within individual countries. Such cross-border cooperations offer valuable research opportunities and are essential to overcome existing hurdles and advance telemedicine in cardiology.

## Abbreviations

AF: Atrial fibrillation
AI: Artificial intelligence
Apps: Applications
CIED: Cardiac implantable electronic device
CRT: Cardiac resynchronisation therapy
CRT‑D: CRT with defibrillator
CRT‑P: CRT pacemaker
CVD: Cardiovascular disease
DICOM: Digital imaging and communications in medicine
DiGA: Digital health applications
ECG: Electrocardiogram
EPR: Electronic patient records
EU: European Union
G‑BA: Federal Joint Committee
GP: General practitioner
HCP: Healthcare professional
HCTR: Hybrid cardiac telerehabilitation
HF: Heart failure
ICD: Implantable cardioverter defibrillator
ILR: Implantable loop recorder
IoT: Internet of things
LPPR: List of reimbursable products and services
LVEF: Left ventricular ejection fraction
NHS: National Health Service
NYHA class: New York Heart Association classification
PAZ: Health application portfolio
Pg/ml: Picograms per millilitre
RPM: Remote patient monitoring
UK: United Kingdom

## Footnotes

1 https://sante.gouv.fr/IMG/pdf/rapport-parlement-novembre2020_v5.pdf, 27.06.2024.

2 https://www.ameli.fr/medecin/exercice-liberal/facturation-remuneration/consultations-actes/nomenclatures-codage/liste-produits-prestations-lpp, 27.06.2024.

3 https://www.g-ba.de/downloads/39-261-4648/2020-12-17_MVV-RL_Telemonitoring-Herzinsuffizienz_BAnz.pdf, 27.06.2024.

4 https://www.movistar.es/salud/telemedicina-medico-online, 26.07.2024.

5 https://puc.overheid.nl/nza/doc/PUC_720738_22/, 13.08.2024.

6 https://www.england.nhs.uk/atlas_case_study/early-intervention-practitioners-in-care-homes/, 27.06.2024.

7 https://www.gov.uk/government/publications/elective-recovery-taskforce-implementation-plan/elective-recovery-taskforce-implementation-plan#:~:text=The%2520Elective%2520Recovery%2520Taskforce%2520was%2520set%2520up%2520in%2520December%25202022,possible%2520to%2520tackle%2520the%25, 27.06.2024.

8 https://www.england.nhs.uk/blog/managing-heart-failure-home-an-opportunity-for-excellence/#:~:text=People%20living%20with%20heart%20failure,failure%20care%20is%20a%20priority., 27.06.2024.

9 https://www.england.nhs.uk/2023/10/nhs-delivers-10000-virtual-ward-beds-target-with-hundreds-of-thousands-of-patients-treated-at-home/, 27.06.2024.

10 https://www.england.nhs.uk/wp-content/uploads/2014/10/5yfv-web.pdf, 27.06.2024.

11 https://www.bfarm.de/SharedDocs/Downloads/DE/Medizinprodukte/diga_leitfaden.html, 27.06.2024.

12 https://eur-lex.europa.eu/legal-content/EN/TXT/?uri=CELEX%3A32017R0745, 27.06.2024.

13 https://www.escardio.org/The-ESC/Press-Office/Press-releases/EP-Societies-Announce-Worldwide-Coalition-to-Improve-CIED-Follow-up-at-Heart-Rhythm-2023-in-New-Orleans, 27.06.2024.

14 https://health.ec.europa.eu/ehealth-digital-health-and-care/european-health-data-space_en, 27.06.2024.

## Declarations

For this article no studies with human participants or animals were performed by any of the authors. All studies mentioned were in accordance with the ethical standards indicated in each case.
